# Influences of elevational gradient on flower size and number of *Gentiana lawrencei* var. *farreri*

**DOI:** 10.1002/ece3.11393

**Published:** 2024-05-13

**Authors:** Mengyan Wang, Zuoyi Wang, Yuan Yang, Xinquan Zhao, Huakun Zhou, Shurong Zhou, Xin Yin, Yanmei Ren, Huanhuan Dong, Longxin Zhang, Zhen Ma, Chunhui Zhang

**Affiliations:** ^1^ State Key Laboratory of Plateau Ecology and Agriculture Qinghai University Xining Qinghai China; ^2^ Key Laboratory of Restoration Ecology for Cold Regions in Qinghai, Northwest Institute of Plateau Biology Chinese Academy of Sciences Xining Qinghai China; ^3^ College of eco‐Environmental Engineering Qinghai University Xining Qinghai China; ^4^ Key Laboratory of Adaptation and Evolution of Plateau Biota, Northwest Institute of Plateau Biology Chinese Academy of Sciences Xining Qinghai China; ^5^ Key Laboratory of Genetics and Germplasm Innovation of Tropical Special Forest Trees and Ornamental Plants, Ministry of Education, College of Forestry Hainan University Haikou Hainan China; ^6^ Qinghai Haibei National Field Research Station of Alpine Grassland Ecosystem, Northwest Institute of Plateau Biology Chinese Academy of Sciences Xining Qinghai China

**Keywords:** alpine plant, climate change, functional trait, individual size, reproductive allocation

## Abstract

Plants can adapt to environmental changes by adjusting their functional traits and biomass allocation. The size and number of flowers are functional traits related to plant reproduction. Life history theory predicts that there is a trade‐off between flower size and number, and the trade‐off can potentially explain the adaptability of plants. Elevation gradients in mountains provide a unique opportunity to test how plants will respond to climate change. In this study, we tried to better explain the adaptability of the alpine plant *Gentiana lawrencei* var. *farreri* in response to climate change. We measured the flower size and number, individual size, and reproductive allocation of *G. lawrencei* var. *farreri* during the flowering period along an elevation gradient from 3200 to 4000 m, and explored their relationships using linear mixed‐effect models and the structural equation model. We found that with elevation increasing, individual size and flower number decreased and flower size increased, while reproductive allocation remained unchanged. Individual size positively affected flower number, but was not related to flower size; reproductive allocation positively affected flower size, but was not related to flower number; there is a clear trade‐off between flower size and number. We also found that elevation decreased flower number indirectly via directly reducing individual size. In sum, this study suggests that *G. lawrencei* var. *farreri* can adapt to alpine environments by the synergies or trade‐offs among individual size, reproductive allocation, flower size, and flower number. This study increases our understanding of the adaptation mechanisms of alpine plants to climate change in alpine environments.

## Graphical abstract

The flower size of *Gentiana lawrencei* var. *farreri* depended on reproductive allocation rather than individual plant size, while the flower number depended on individual plant size rather than reproductive allocation. There was a clear trade‐off between flower size and number in *G. lawrencei* var. *farreri*, and individual size partially masked this trade‐off.

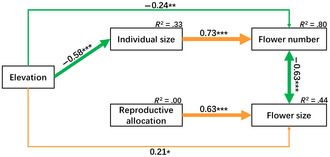

## 1 INTRODUCTION

Plants can adapt to environmental changes by adjusting their functional traits and reproductive allocation (Atkin et al., 2015; Körner, 2003). Floral display in terms of flower size and number has influences on pollinator visitation rate and seed production (Bell, 1985; Sandring & Ågren, 2009; Wu et al., 2023). Therefore, floral display is an important reproductive trait related to fitness (Sargent et al., 2007), and is often used to study plant evolution and adaptation mechanisms (Sandring & Ågren, 2009; Zhang et al., 2017). Plants have evolved diverse flower displays to adapt to environmental changes and extreme environments (such as high‐altitude environments) (Körner, 2003; Kuppler & Kotowska, 2021; Zhang et al., 2017).

Life history theory suggests that a plant allocates its resources to different needs, which lead to trade‐offs between different functions and activities due to finite resources of a plant (Silvertown & Charlesworth, 2009). That is to say, an increase in the amount of resources invested in a certain function (trait) will inevitably lead to a decrease in the amount of resources invested in other functions (traits). Reproductive allocation controls the balance between plant survival and reproduction, and is one of the core theories of plant life history (Friedman, 2020). Plants tend to choose optimal reproductive allocation to achieve maximum fitness (Wong & Ackerly, 2005). Non‐biological (e.g., elevation) and biological (e.g., competition) factors are generally believed to shift the balance between vegetative growth and reproduction (Friedman, 2020). In addition, within reproduction function, there should be a trade‐off between flower size and number based on hierarchical resource allocation (Obeso, 2002; Richards et al., 2009). That is to say, plants can either produce fewer and larger flowers or produce more and smaller flowers. Life history theory also suggests that the evolution of floral display is constrained by the trade‐off between flower size and number (Sargent et al., 2007). Although its existence has a convincing theoretical basis, empirical evidence of the trade‐off between flower size and number has always been elusive (reviewed by Caruso et al., 2012 and Sargent et al., 2007). For instance, some empirical studies have found no relationship between flower size and number (Ashman & Majetic, 2006; Caruso et al., 2012; Worley & Barrett, 2001).

**FIGURE 1:**
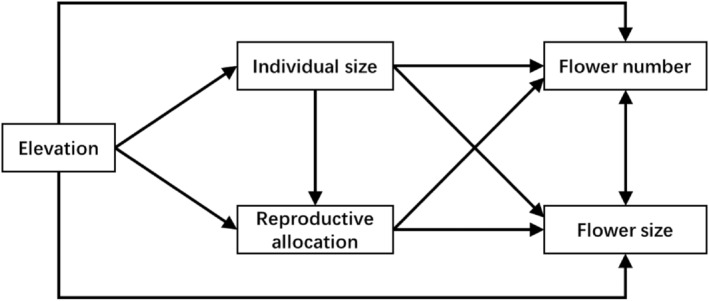
The hypothetical pathways of how elevation influences flower size and number through individual size and reproductive allocation.

Elevation gradients in mountains are usually used as a substitution to test how plants will respond to climate change (Li et al., 2016). High‐altitude areas have harsh environments, such as low temperatures, short growing season, and variability and poor predictability of climate, which not only affect the growth and development of alpine plants but also affect the interaction between plants and animals (Körner, 2003). Due to unfavorable growth conditions, plant size generally decreases with increasing elevation (Coomes & Allen, 2007; Kiełtyk, 2021; Sigdel et al., 2023). It is generally believed that in high‐altitude environments, plant reproduction and population persistence suffer greater pressure, resulting in an increase in plant reproductive allocation as elevation increases (Fabbro & Körner, 2004; Rathee et al., 2021). The individual size of plants can reflect the total amount of plant resources. The increase in both total plant resources and reproductive allocation can potentially increase the number and size of flowers (Zhang, 2004). One might predict then that elevation could indirectly influence flower size and number via individual size and reproductive allocation (Figure 1).

Numerous researches have shown that the diversity and abundance of pollinating insects and the number of flower‐visiting insects per plant decreased with increasing elevation (Adedoja et al., 2020; Lefebvre et al., 2018). Compared to low‐altitude areas, lower temperatures in high‐altitude environments can weaken the activity of flower‐visiting insects (Arroyo et al., 1985; Bingham & Ortner, 1998; Goodwin et al., 2021). Therefore, many outcrossing alpine plants may suffer from strong pollination limitation (Duan et al., 2007; Sun et al., 2018), which may reduce the success rate of outcrossing plant reproduction. In order to adapt to the special environment of alpine regions and attract more insects to visit flowers, plants must make some efforts to potentially produce larger flowers (Kiełtyk, 2018; Körner, 2003).

*Gentiana* species are typical alpine plants, with the Qinghai‐Tibet Plateau and its surrounding areas as their distribution and differentiation centers (Ho & Liu, 2001). As a common species of the *Gentiana* genus, *Gentiana lawrencei* var. *farreri* is a perennial herbaceous plant endemic to the Qinghai‐Tibet Plateau, a dominant species in alpine meadows (Ho & Liu, 2001) and an important traditional medicinal plant (Yang et al., 1991). Elevation is the key determinant of bioclimatic gradients on the Qinghai‐Tibet Plateau (Qi et al., 2015; Sigdel et al., 2023; Zhang et al., 2022). The unique alpine environmental conditions of the Qinghai‐Tibet Plateau provide a relatively good platform for studying the adaptability and evolutionary mechanisms of plant resource allocation (Duan et al., 2007; Hou et al., 2024). Here, we chose the typical alpine plant *G. lawrencei* var. *farreri* in the Qinghai‐Tibet Plateau as the research object, mainly studying the relationship of flower size and number, as well as the effects of elevation, individual size, and plant reproductive allocation on flower size and number. We hypothesized that elevation could influence flower size and number directly and indirectly via individual size and reproductive allocation (Figure 1). To our knowledge, there is no published research on the direct and indirect effect of elevation on flower size and number. Specifically, the objectives of our study were to determine: (1) the direct and indirect effect of elevation on flower size and number through its direct impacts on individual size and reproductive allocation, and (2) whether there was a trade‐off between flower size and number (Figure 1).

## 2 MATERIALS AND METHODS

### 2.1 Study area

The experiment was carried out at the Qinghai Haibei National Field Research Station of Alpine Grassland Ecosystem in China (37°37′ N, 101°12′ E). The station is situated in the northeast of the Qinghai‐Tibet Plateau. The region has a typical plateau continental climate, with long and cold winters and short and cool summers (Zhang et al., 2019). In September 2023, we sampled *G. lawrencei* var. *farreri* during flowering every 100–300 m along a 3200–4000 m elevational gradient (Table S1; see photographs of the habitat of *G. lawrencei* var. *farreri* in Figure S1) on the south slope of the Qilian Mountains in Qinghai, China. A long‐term field study (Li et al., 2016) in the same site reported that annual average soil temperatures at 5 cm depth were 3.9, 2.5, 2.0, and 0.4 °C, and annual average soil moistures at 20 cm depth were 11.8, 11.3, 12.7, and 10.2% at 3200, 3400, 3600, and 3800 m, respectively.

### 2.2 Study species

*G. lawrencei* var. *farreri* is a perennial herbaceous plant with a height of 5–12 cm. The flower branches are numerous, clustered, and scattered. The rosette leaves are extremely underdeveloped and lanceolate, with a length of 4–6 mm and a width of 2–3 mm. One flower grows at the top of the branch, and is inverted cone‐shaped, with a sky‐blue upper part and a yellow green lower part, and with blue stripes and a length of 4.5–6.0 cm. It is mainly distributed in the northeastern Qinghai‐Tibetan Plateau from 2400 to 4000 m above sea level (Ho & Liu, 2001). Its peak flowering period is in mid‐September, and its main pollinating insects are *Bombus kashmirensis* and *B. sushikini* (Hou et al., 2009).

### 2.3 Sampling and data measurement

We selected the *G. lawrencei* var. *farreri* population that met the sampling requirements (including at least 30 healthy, fully developed flowering individuals) every 100–300 m along the elevational gradient. We then randomly selected 12 apparently healthy, fully developed flowering individuals from the population of *G. lawrencei* var. *farreri* at each elevation (3200, 3500, 3750, 3900, and 4000 m above sea level). We counted the number of flowers per individual, then divided each individual into flowers and the rest, dried them at 60°C for 48 h, and weighed them. Based on these data, we obtained the data of individual size (aboveground biomass), reproductive allocation (total flower weight/aboveground biomass), and flower size (total flower weight/number of flowers) and number (the number of flowers per individual).

### 2.4 Data analyses

We applied standard linear models to examine the relationships between elevation and individual size, reproductive allocation, flower size, and flower number.

The relationships of individual size with reproductive allocation and total flower weight (i.e., the absolute amount of reproduction) were tested using linear mixed‐effect models, respectively. Linear mixed‐effect models were also used to examine the effects of individual size and reproductive allocation on flower size and number, respectively. The plant population was used as a random factor in these models.

We conducted a linear mixed‐effect model to detect the relationship between flower size and number. The differences in plant resource acquisition ability potentially caused by different individual sizes may affect the trade‐off between flower size and number. Therefore, we use residuals to control the changes in individual size. Specifically, the residuals of flower size on individual size and the residuals of flower number on individual size were used in bivariate regressions to examine the relationship between flower size and number, independent of variation in individual size, using the linear mixed‐effect model. The plant population was used as a random factor in these models.

We used the structural equation model (SEM) to explore the connections among elevation, individual size, reproductive allocation, flower size, and flower number. We hypothesized a path diagram where elevation regulates flower size and number directly or indirectly through individual size and reproductive allocation (Figure 1). The outputs of the SEM were produced based on the *p*‐values of conditional independence tests combined into a single Fisher's C statistic.

The standard linear model, linear mixed‐effect model and SEM were conducted using the packages base (Tollefson, 2019), lmerTesT (Bates et al., 2015), and piecewiseSEM (Lefcheck, 2016) in R environment (R Core Team, 2023), respectively.

## 3 RESULTS

### 3.1 Relationships between elevation and individual size, reproductive allocation, flower size, and flower number

**FIGURE 2:**
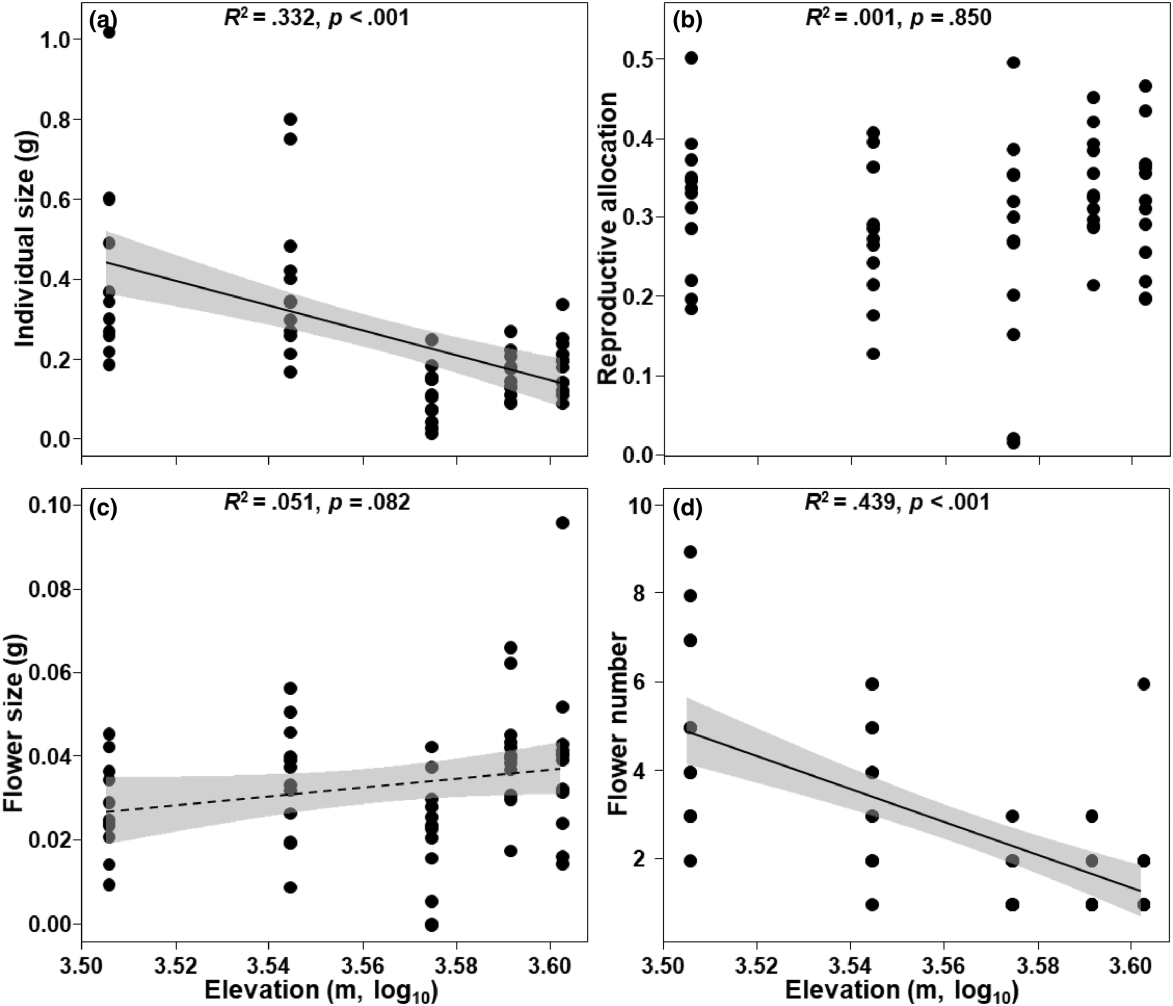
Relationships between elevation and individual size (a), reproductive allocation (b), flower size (c), and flower number (d).

With elevation increasing, individual size (*p* < .001; Figure 2a) and flower number (*p* < .001; Figure 2d) significantly decreased, while flower size marginally significantly increased (*p* = .082; Figure 2c), while reproductive allocation did not show significant changes (*p* = .850; Figure 2b).

### 3.2 Relationships of individual size and reproductive allocation with flower size and number

**FIGURE 3:**
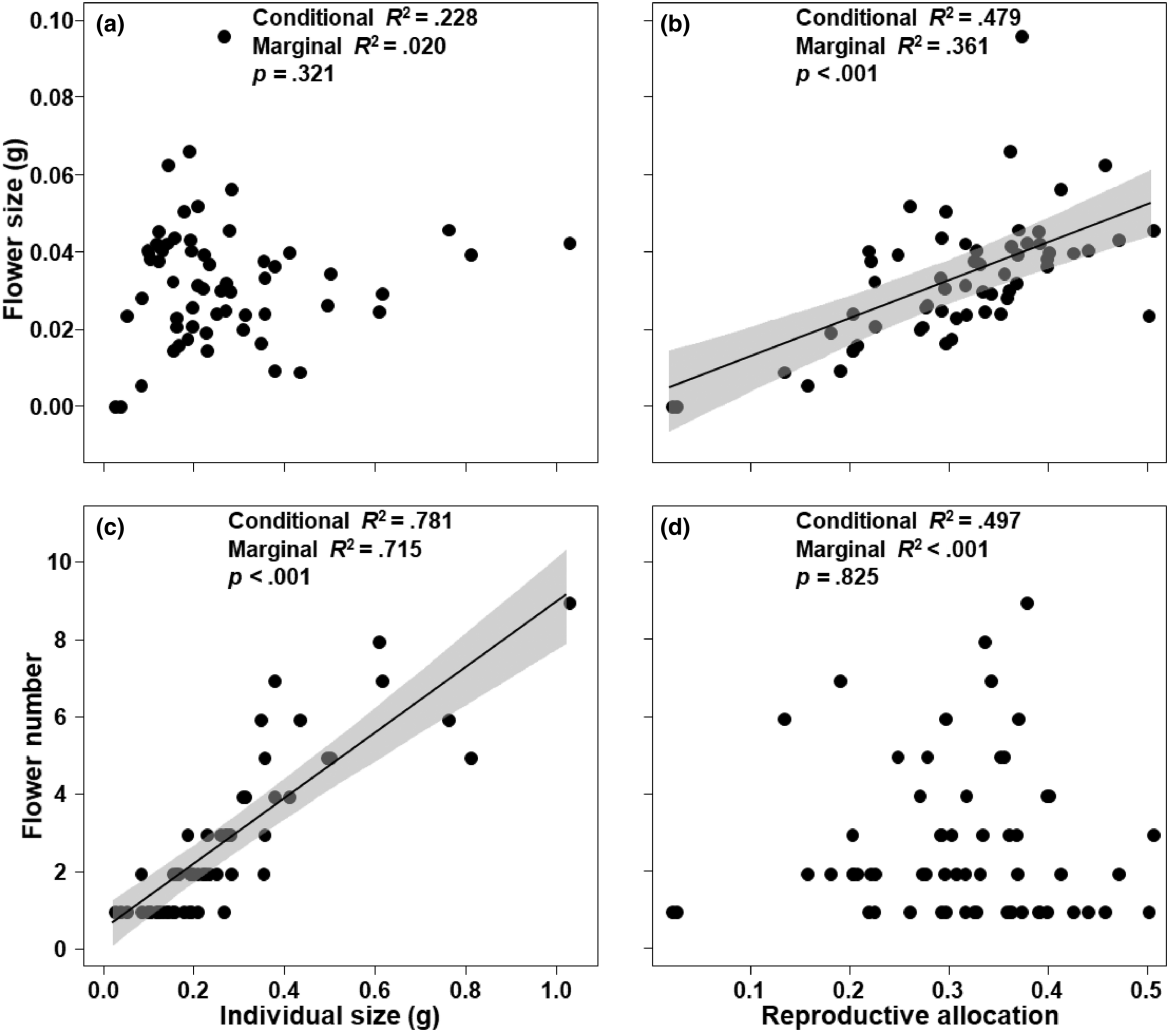
Relationships of individual size and reproductive allocation with flower size (a, b) and number (c, d). Residuals were used to control the changes in individual size. The residuals of flower size on individual size (i.e., residuals flower size) and the residuals of flower number on individual size (i.e., residuals flower number) were used in bivariate regressions to examine the relationship between flower size and number, independent of variation in individual size, using the linear mixed‐effect model.

We found that individual size significantly positively affected flower number (*p* < .001; Figure 3c), but had no effect on flower size (*p* = .321; Figure 3a). Reproductive allocation significantly positively affected flower size (*p* < .001; Figure 3b), but had no effect on flower number (*p* = .825; Figure 3d).

### 3.3 The relationships of individual size with reproductive allocation and total flower weight

We found that individual size had no effect on reproductive allocation (*p* = .486; Figure S2a). The was a significant relationship between individual size and total flower weight (*p* < .001; Figure S2b).

### 3.4 The trade‐off between flower size and number

**FIGURE 4:**
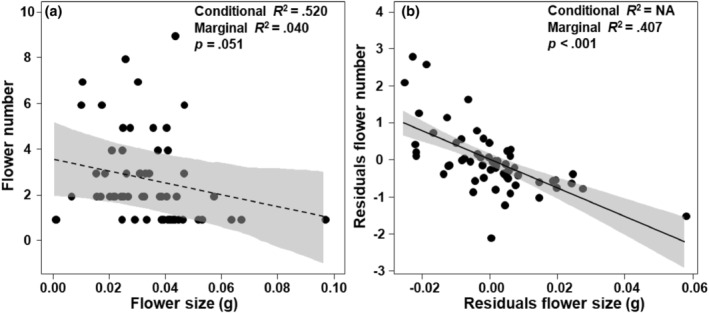
The trade‐off between flower size and number when not controlling (a) and controlling (b) for variation in individual size. Residuals were used to control the changes in individual size. The residuals of flower size on individual size (i.e., residuals flower size) and the residuals of flower number on individual size (i.e., residuals flower number) were used in bivariate regressions to examine the relationship between flower size and number, independent of variation in individual size, using the linear mixed‐effect model.

We found that there was a marginally significantly negative correlation between flower size and number in *G. lawrencei* var. *farreri* (*p* = .051; Figure 4a). When controlling for variation in individual size, there was a significant trade‐off between flower size and number (*p* < .001; Figure 4b).

### 3.5 Results of the SEM

**FIGURE 5:**
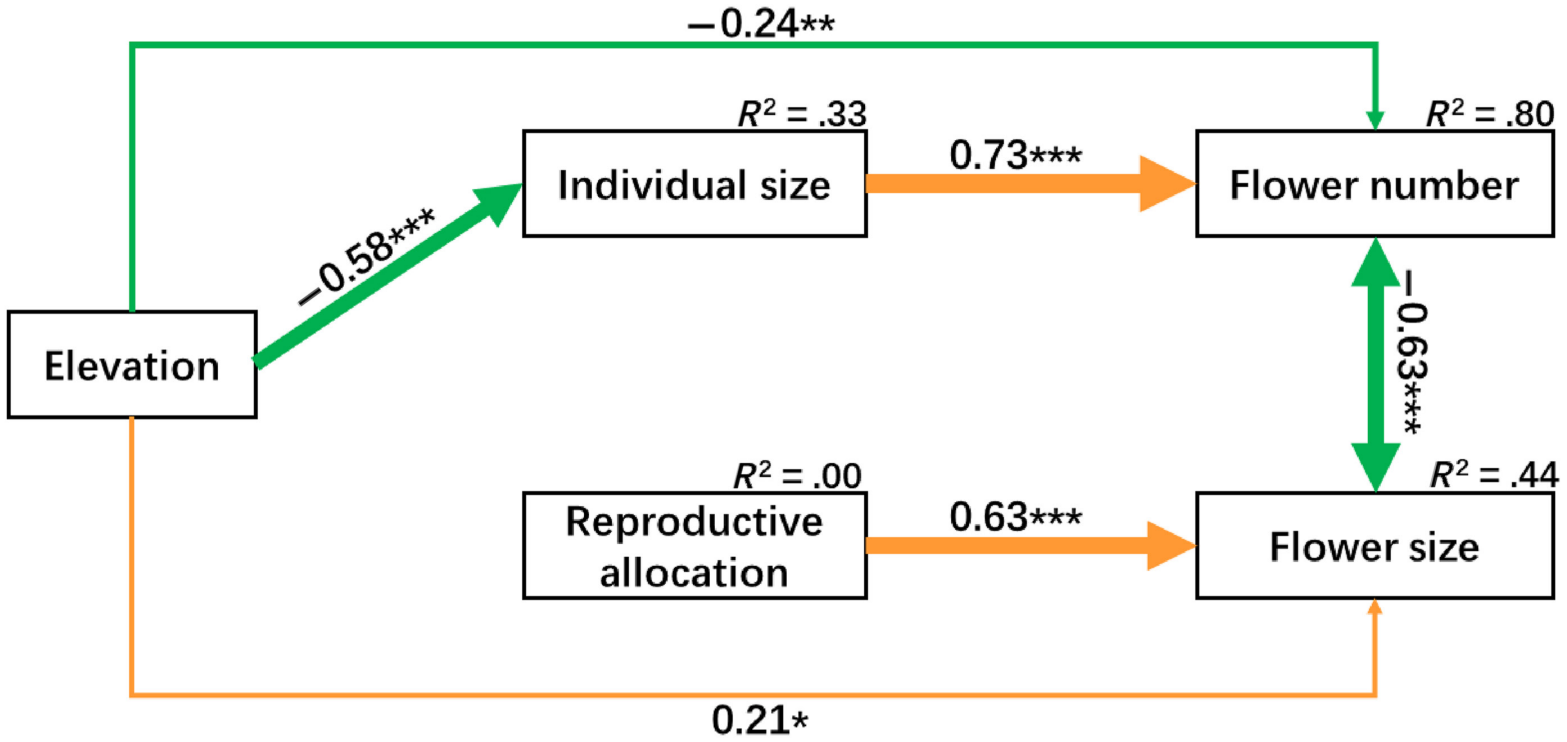
Structural equation model (SEM) of the relationships among elevation, individual, reproductive allocation, flower size, and flower number. Solid yellow and green arrows indicate significant negative and positive effects (*p < .05; **p < .01; ***p < .001), respectively. The numbers above arrows indicate path coefficients. The width of arrows indicates the strength of the causal influence. *R* ^2^ represents the proportion of variance explained for each dependent variable in the model (Fisher's C = 10.55, *p* = .103, *df* = 6).

According to the parameter values, the SEM adequately fitted the data (Fisher's C = 10.551, *p* = .103, *df* = 6). The results of the SEM (Figure 5) showed that: (1) there was a significant negative correlation between flower size and number; (2) individual size directly and significantly affected flower number, while reproductive allocation directly and significantly affected flower size; (3) elevation negatively affected flower number directly or indirectly through individual size; (4) elevation directly affected flower size. These results were consistent with the results of linear models (Figures 2, 3, and 4). In addition, the SEM explained 33%, 0%, 44%, and 80% variance in individual size, reproductive allocation, flower size, and flower number, respectively.

## 4 DISCUSSIONS

We explored the variation patterns in individual size, reproductive allocation, flower size, and flower number, as well as their relationships, of a typical alpine plant, *G. lawrencei* var. *farreri*, on an elevation gradient of 3200–4000 m in the northeast of the Qinghai‐Tibet Plateau. We found that with elevation increasing, the individual size of *G. lawrencei* var. *farreri* became smaller, and the flowers became larger and fewer, while the reproductive allocation remained unchanged. There was a trade‐off between flower size and number in *G. lawrencei* var. *farreri*, which reflects its adaptation to alpine biotic and abiotic environments.

Plant vegetative growth and reproduction promote and restrict each other, and their response to environmental changes depends on the investment balance of plants in vegetative growth and reproduction (Zhang, 2004). The accumulated biomass of *G. lawrencei* var. *farreri* decreased sharply with increasing elevation, due to harsh environments (e.g., low temperatures and variability and poor predictability of climate) at high‐altitude areas (Körner, 2003; Zhang et al., 2020). This is consistent with most experimental and theoretical research results (Coomes & Allen, 2007; Kiełtyk, 2021; Körner, 2003; Sigdel et al., 2023). We found that the reproductive allocation of *G. lawrencei* var. *farreri* was relatively constant and did not increase or decrease with changes in elevation. This is inconsistent with most empirical and theoretical studies which suggested plant reproductive allocation increased with elevation (Fabbro & Körner, 2004; Rathee et al., 2021). A previous study showed a significant positive correlation between reproductive allocation and elevation for annual and biennial plants, while the correlation between the two was not significant in perennial plants (Zhang et al., 2020). For perennial plants (e.g., *G. lawrencei* var. *farreri*), the trade‐off between current and future reproduction may mask the trade‐off between vegetative growth and reproductive growth (Zhang, 2004). In addition, as elevation increases, some ecological factors may improve (e.g., soil organic carbon often accumulating in large stocks in cold regions; García‐Palacios et al., 2024), which may result in reproductive allocation not showing an upward trend with elevation, or even showing a downward trend. Finally, this study found that there was a trade‐off between flower size and number in *G. lawrencei* var. *farreri*, which may also replace the trade‐off between vegetative growth and reproduction to some extent, in order to adapt to high‐altitude environmental conditions (see later discussion).

We also found that the reproductive allocation of *G. lawrencei* var. *farreri* did not increase or decrease with changes in individual size. This is inconsistent with previous empirical and theoretical studies (reviewed by Zhang & Jiang, 2002 and Zhang, 2004). These previous studies suggested that the larger the individual, the smaller the resources invested in the reproductive part. The decrease in reproductive allocation with increasing individual size may be a direct result of the increase in reproductive costs which can be partially explained by an increase in the allocation of reproductive support structures with increasing individual size (Reekie, 1998). In our study, the relatively smaller individuals of *G. lawrencei* var. *farreri* tended to grow at higher elevations, thus facing greater potential risks and resulting in higher reproductive costs. This may potentially lead to results in this study that reproductive allocation was not related to individual size.

The flower size of entomophilous plants affects plant attractiveness to pollinators (Krizek & Anderson, 2013; Teixido et al., 2018), which in turn affects plant reproductive success (Hou et al., 2024; Wei et al., 2021). In alpine environments, as elevation increases, environmental variables such as temperature, growth season length, and resource availability decrease, and the diversity, abundance, and activity ability of pollinating insects also decrease accordingly (Körner, 2003). The shortage of pollinators is the main selection pressure for the evolution of reproductive strategies in alpine plants (Duan et al., 2007; Sun et al., 2018). In high‐altitude environments, plants may potentially increase their investment in male organs or attraction structures (Fabbro & Körner, 2004; Rathee et al., 2021), thereby reducing the impact of adverse factors such as the scarcity of pollinators, which is beneficial for improving plant pollination success rate. This may be the potential reason why we found that the flower size of the *G. lawrencei* var. *farreri* increased with elevation increasing. Meanwhile, we also found that the flower number of the *G. lawrencei* var. *farreri* decreased with elevation increasing. Therefore, the changes in biotic (pollination limitation) and abiotic (temperature and resource availability decreasing) environments along the elevation gradient jointly derived the trade‐off between flower size and number in *G. lawrencei* var. *farreri* (Figure 4). This trade‐off is of great significance in plant evolution and adaptation (Caruso et al., 2012; Sargent et al., 2007). In this study, the trade‐off between flower size and number may potentially improve the adaptability of plants to climate change along an elevation gradient. The empirical evidence for this trade‐off is mixed (reviewed by Caruso et al., 2012 and Sargent et al., 2007). We found that the differences in plant resource acquisition ability potentially caused by individual size may mask the true trade‐off relationship (Figure 4).

An interesting finding in this study is that the flower size of *G. lawrencei* var. *farreri* depended on reproductive allocation rather than individual plant size, and in contrast, flower number depended on individual plant size rather than reproductive allocation (Figure 3). That is to say, when plant reproduction allocation increased (i.e., the relative amount of reproduction increasing), plants tended to invest in flower size but not in flower number; when the individual size of a plant increased (equivalent to an increase in the absolute amount of reproduction; Figure S2b), the plant tended to invest in flower number but not in flower size. It is generally believed that the size and number of flowers increase with individual size, and previous studies have confirmed this point (for flower size: Worley et al., 2000; Worley & Barrett, 2001; for flower number: Morgan, 1998; Sato & Yahara, 1999; Sun et al., 2018; Worley & Barrett,  2000). However, similar to several previous studies (Iwaizumi & Sakai, 2004; Sato & Yahara, 1999), we found that flower size did not increase with individual size. It might be explained by the trade‐off between flower size and number, as well as the evidence that we found that individual size can increase the number of flowers. Through multiple statistical methods such as linear mixed‐effect and structural equation model analyses, we found that flower size was controlled by the relative reproductive allocation rather than the absolute reproductive allocation, while flower number was controlled by the absolute reproductive allocation rather than the relative reproductive allocation. Our results provided a new perspective for this research question. Of course, more researches will be needed in the future to validate our results.

## 5 CONCLUSIONS

We found that along an elevation of 3200–4000 m, *G. lawrencei* var. *farreri* became smaller and its flowers became larger and fewer, while the reproductive allocation remained unchanged. The flower size of *G. lawrencei* var. *farreri* depended on reproductive allocation rather than individual plant size, while the flower number depended on individual plant size rather than reproductive allocation. There was a clear trade‐off between flower size and number in *G. lawrencei* var. *farreri*, and individual size partially masked this trade‐off. Elevation decreased flower number directly and indirectly via reducing individual size. And elevation and reproductive allocation both directly increased flower size. The variation patterns in individual size, reproductive allocation, flower size, and flower number of *G. lawrencei* var. *farreri*, as well as the trade‐off between flower size and number, reflect its adaptation to alpine environments. This study increases our understanding of the adaptation mechanisms of alpine plants to climate change on the elevation gradient.

## AUTHOR CONTRIBUTIONS

**Mengyan Wang:** Data curation (equal); investigation (equal); methodology (equal); software (equal); writing – original draft (equal). **Zuoyi Wang:** Data curation (equal); investigation (equal); methodology (equal); visualization (equal); writing – review and editing (equal). **Yuan Yang:** Data curation (equal); investigation (equal); visualization (equal); writing – review and editing (equal). **Xinquan Zhao:** Funding acquisition (equal); project administration (equal); resources (equal); writing – review and editing (equal). **Huakun Zhou:** Methodology (equal); resources (equal); writing – review and editing (equal). **Shurong Zhou:** Methodology (equal); resources (equal); writing – review and editing (equal). **Xin Yin:** Methodology (equal); software (equal); writing – review and editing (equal). **Yanmei Ren:** Investigation (equal); methodology (equal); visualization (equal); writing – review and editing (equal). **Huanhuan Dong:** Investigation (equal); writing – review and editing (equal). **Longxin Zhang:** Investigation (equal); writing – review and editing (equal). **Zhen Ma:** Conceptualization (equal); methodology (lead); project administration (equal); supervision (equal); writing – original draft (equal). **Chunhui Zhang:** Conceptualization (lead); formal analysis (equal); funding acquisition (lead); methodology (equal); project administration (equal); supervision (lead); writing – original draft (lead).

## CONFLICT OF INTEREST STATEMENT

The authors declare that they have no conflict of interest.

## Supporting information


Tables S1–S2.
Figures S1–S2.

## Data Availability

The data that support our paper can be found in Supporting Information.
