# Supplementary material for: Influences of elevational gradient on flower size and number of Gentiana lawrencei var. farreri
Source: Ecol Evol. 2024 May 13;14(5):e11393. doi: 10.1002/ece3.11393 (PMC11091485; doi:10.1002/ece3.11393)
Supplement: Supplementary file 1 — Tables S1–S2. Figures S1–S2. [file ECE3-14-e11393-s001.doc]

**Supporting Information**

TABLE S1 The longitude and latitude at each elevation

| **NO** | **Elevation** | **Longitude** | **Latitude** |
| --- | --- | --- | --- |
| **1** | 3200 | 101.2995 | 37.6211 |
| **2** | 3500 | 101.3556 | 37.6867 |
| **3** | 3750 | 101.3682 | 37.7031 |
| **4** | 3900 | 101.3698 | 37.7061 |
| **5** | 4000 | 101.3726 | 37.7080 |

TABLE S2 Data of this study

| **Elevation** | **Total flower weight** | **Flower number** | **Flower size** | **Individual size** | **Reproductive allocation** |
| --- | --- | --- | --- | --- | --- |
| **3200** | 0.0766 | 3 | 0.025533 | 0.2647 | 0.289384 |
| **3200** | 0.1225 | 5 | 0.0245 | 0.3501 | 0.3499 |
| **3200** | 0.3859 | 9 | 0.042878 | 1.0243 | 0.376745 |
| **3200** | 0.1477 | 4 | 0.036925 | 0.373 | 0.395979 |
| **3200** | 0.0448 | 3 | 0.014933 | 0.2236 | 0.200358 |
| **3200** | 0.0428 | 2 | 0.0214 | 0.1918 | 0.223149 |
| **3200** | 0.2078 | 7 | 0.029686 | 0.6106 | 0.340321 |
| **3200** | 0.0969 | 4 | 0.024225 | 0.3074 | 0.315224 |
| **3200** | 0.1754 | 5 | 0.03508 | 0.4965 | 0.353273 |
| **3200** | 0.2017 | 8 | 0.025213 | 0.6049 | 0.333444 |
| **3200** | 0.138 | 3 | 0.046 | 0.2738 | 0.504018 |
| **3200** | 0.0702 | 7 | 0.010029 | 0.3741 | 0.18765 |
| **3500** | 0.0395 | 2 | 0.01975 | 0.2209 | 0.178814 |
| **3500** | 0.0814 | 4 | 0.02035 | 0.3035 | 0.268204 |
| **3500** | 0.1987 | 5 | 0.03974 | 0.8074 | 0.246099 |
| **3500** | 0.1137 | 2 | 0.05685 | 0.2771 | 0.410321 |
| **3500** | 0.2781 | 6 | 0.04635 | 0.7574 | 0.367177 |
| **3500** | 0.0761 | 2 | 0.03805 | 0.3477 | 0.218867 |
| **3500** | 0.135 | 5 | 0.027 | 0.4897 | 0.275679 |
| **3500** | 0.0563 | 6 | 0.009383 | 0.4287 | 0.131327 |
| **3500** | 0.1013 | 3 | 0.033767 | 0.3513 | 0.288358 |
| **3500** | 0.0511 | 1 | 0.0511 | 0.1735 | 0.294524 |
| **3500** | 0.1619 | 4 | 0.040475 | 0.406 | 0.398768 |
| **3500** | 0.0973 | 3 | 0.032433 | 0.2661 | 0.365652 |
| **3750** | 0.0472 | 2 | 0.0236 | 0.1551 | 0.30432 |
| **3750** | 0.0285 | 1 | 0.0285 | 0.0801 | 0.355805 |
| **3750** | 0.0241 | 1 | 0.0241 | 0.0483 | 0.498965 |
| **3750** | 0.0122 | 2 | 0.0061 | 0.0788 | 0.154822 |
| **3750** | 0.0005 | 1 | 0.0005 | 0.0216 | 0.023148 |
| **3750** | 0.0522 | 2 | 0.0261 | 0.1906 | 0.273872 |
| **3750** | 0.0381 | 1 | 0.0381 | 0.1178 | 0.32343 |
| **3750** | 0.0424 | 2 | 0.0212 | 0.1567 | 0.270581 |
| **3750** | 0.0912 | 3 | 0.0304 | 0.2546 | 0.358209 |
| **3750** | 0.0327 | 2 | 0.01635 | 0.1601 | 0.204247 |
| **3750** | 0.0006 | 1 | 0.0006 | 0.0329 | 0.018237 |
| **3750** | 0.0428 | 1 | 0.0428 | 0.1101 | 0.388738 |
| **3900** | 0.0457 | 1 | 0.0457 | 0.1178 | 0.387946 |
| **3900** | 0.091 | 3 | 0.030333 | 0.2745 | 0.331512 |
| **3900** | 0.0404 | 1 | 0.0404 | 0.0954 | 0.42348 |
| **3900** | 0.0408 | 1 | 0.0408 | 0.188 | 0.217021 |
| **3900** | 0.063 | 1 | 0.063 | 0.1383 | 0.455531 |
| **3900** | 0.0666 | 1 | 0.0666 | 0.1856 | 0.358836 |
| **3900** | 0.0542 | 3 | 0.018067 | 0.1804 | 0.300443 |
| **3900** | 0.075 | 2 | 0.0375 | 0.2282 | 0.328659 |
| **3900** | 0.0389 | 1 | 0.0389 | 0.0982 | 0.39613 |
| **3900** | 0.0441 | 1 | 0.0441 | 0.152 | 0.290132 |
| **3900** | 0.0426 | 1 | 0.0426 | 0.1357 | 0.313928 |
| **3900** | 0.0626 | 2 | 0.0313 | 0.213 | 0.293897 |
| **4000** | 0.0796 | 2 | 0.0398 | 0.2173 | 0.366314 |
| **4000** | 0.0964 | 1 | 0.0964 | 0.2597 | 0.371198 |
| **4000** | 0.03 | 2 | 0.015 | 0.1496 | 0.200535 |
| **4000** | 0.0492 | 2 | 0.0246 | 0.2447 | 0.201063 |
| **4000** | 0.041 | 1 | 0.041 | 0.0936 | 0.438034 |
| **4000** | 0.0524 | 1 | 0.0524 | 0.2028 | 0.258383 |
| **4000** | 0.1007 | 6 | 0.016783 | 0.3423 | 0.294186 |
| **4000** | 0.0872 | 2 | 0.0436 | 0.1859 | 0.469069 |
| **4000** | 0.0411 | 1 | 0.0411 | 0.1264 | 0.325158 |
| **4000** | 0.0421 | 1 | 0.0421 | 0.117 | 0.359829 |
| **4000** | 0.064 | 2 | 0.032 | 0.2037 | 0.314188 |
| **4000** | 0.0328 | 1 | 0.0328 | 0.1476 | 0.222222 |


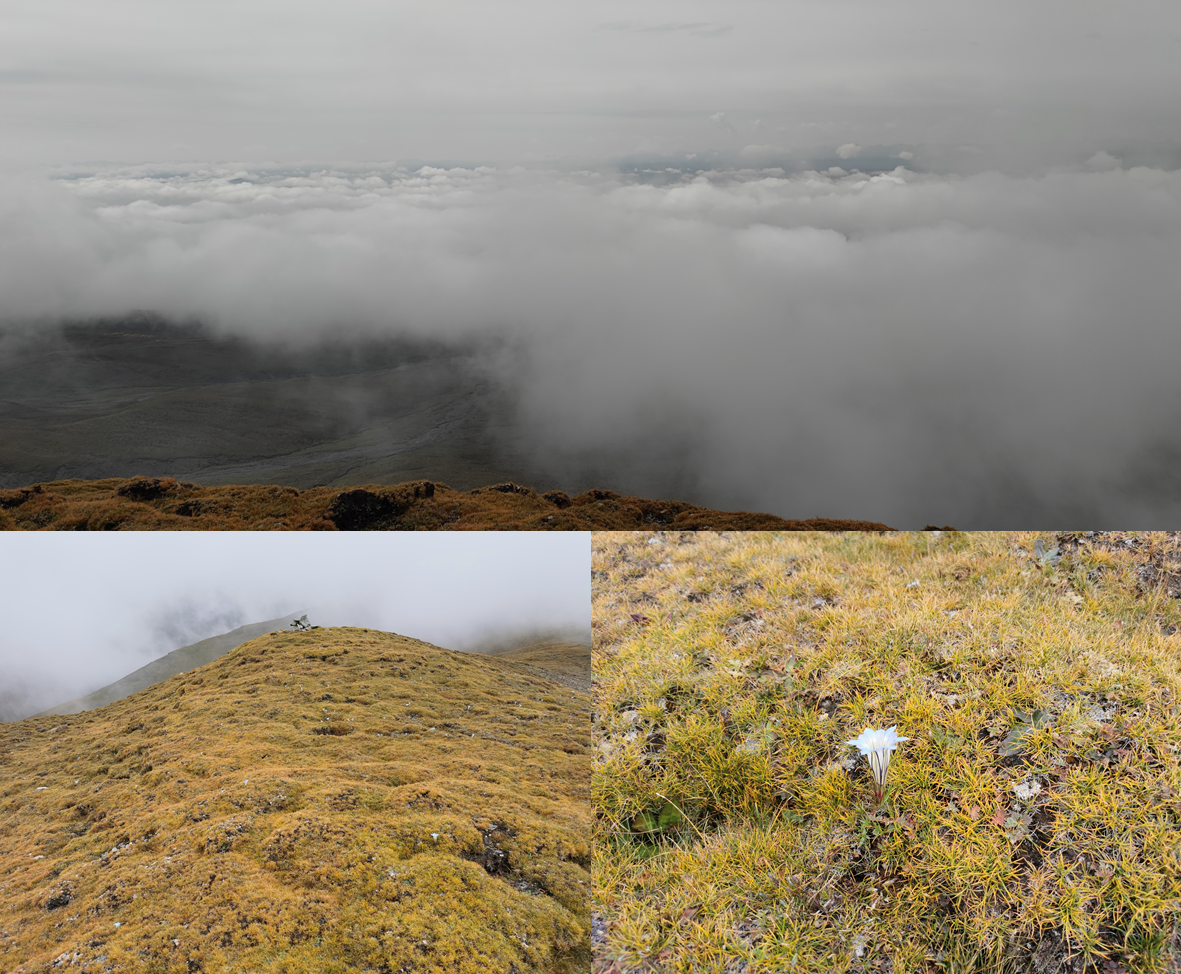


**FIGURE S1** Photographs of the habitat of *G. lawrencei* var. *farreri.*


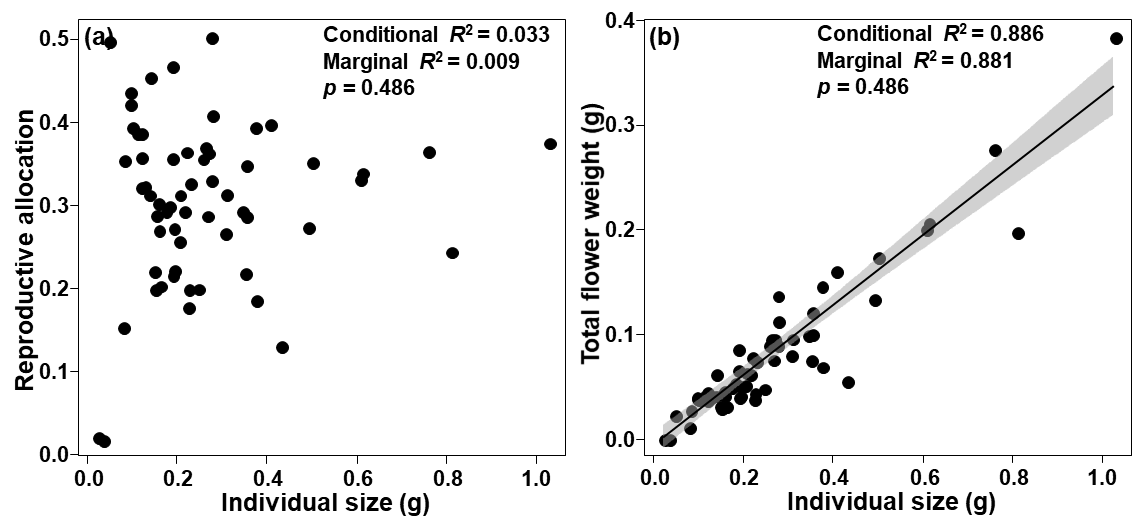


**FIGURE S2** The relationships of individual size with reproductive allocation (a) and total flower weight (b).
